# Avoiding inappropriate paediatric admission: facilitating General Practitioner referral to Community Children’s Nursing Teams

**DOI:** 10.1186/1471-2296-14-4

**Published:** 2013-01-05

**Authors:** Richard G Kyle, Michele Banks, Susan Kirk, Peter Powell, Peter Callery

**Affiliations:** 1 School of Nursing, Midwifery and Health, University of Stirling (Highland Campus), Centre for Health Science, Old Perth Road, Inverness IV2 3JH, UK; 2 School of Nursing, Midwifery and Social Work, The University of Manchester, Manchester Academic Health Science Centre, Jean McFarlane Building, Oxford Road, Manchester, M13 9PL, UK; 3 West Suffolk Hospital, Bury St Edmunds, IP33 2QZ, UK

## Abstract

**Background:**

Children’s emergency admissions in England are increasing. Community Children’s Nursing Teams (CCNTs) have developed services to manage acutely ill children at home to reduce demand for unscheduled care. Referral between General Practitioners (GPs) and CCNTs may reduce avoidable admissions and minimise the psychosocial and financial impact of hospitalisation on children, families and the NHS. However, facilitators of GP referral to CCNTs are not known. The aim of this study was to identify facilitators of GP referral to CCNTs.

**Methods:**

Semi-structured interviews with 39 health professionals were conducted between June 2009 and February 2010 in three Primary Care Trusts served by CCNTs in North West England. Interviewees included GPs, Community Children’s Nurses (CCNs), consultant paediatricians, commissioners, and service managers. Qualitative data were analysed thematically using the Framework approach in NVivo 8.

**Results:**

Five facilitators were identified: 1) CCN/CCNT visibility; 2) clear clinical governance procedures; 3) financial and organisational investment in the role of CCNTs in acute care pathways; 4) access and out of hours availability; 5) facilitative financial frameworks.

**Conclusion:**

GPs required confidence in CCNs’ competence to safely manage acutely ill children at home and secure rapid referral if a child’s condition deteriorated. Incremental approaches to developing GP referral to CCNTs underpinned by clear clinical governance protocols are likely to be most effective in building GP confidence and avoiding inappropriate admission.

## Background

General Practitioners (GPs) have been described as the main healthcare providers for children and young people [1]. GPs are the first point of contact for many parents of children with acute conditions [2,3], and their knowledge of the whole family has the potential to avoid ‘inappropriate’ referrals to paediatricians [4]. Children’s emergency admissions in England have continued to increase [5,6] despite overall improvements in children’s well-being [7]. Concerns have been raised that this trend may indicate parents bypass primary care when seeking care for their acutely ill child [8], perhaps due to availability of out of hours services. However, there is also evidence that parents of febrile children make appropriate judgements and initially approach their GP, and that multiple contacts are often initiated by services with undetermined benefit to children’s care [9]. The option of referral to Community Children's Nursing Teams (CCNTs) that can provide care to children at home by nurses with paediatric training has the potential to avoid some onward referrals and preventable admissions. CCNTs have been found to be acceptable to parents and children [10,11], although there is limited evidence about the clinical- [12] and cost-effectiveness of paediatric home care [13]. Care at home that prevents avoidable attendances at Emergency Departments (ED), or even hospitalisation, could minimise the psychosocial and financial costs for children, families and the National Health Service (NHS). However, relationships between GPs and CCNTs, including what factors affect GPs’ understanding of CCNTs and practices in referral to CCNT services are poorly understood [14] and factors facilitative of GP referral to CCNTs are not known. The aim of this study was therefore to identify facilitators of GP referral to CCNTs.

## Methods

### Design

Qualitative comparative case studies were conducted of three purposively sampled CCNTs serving three Primary Care Trusts (PCTs) in North West England. Purposive sampling was conducted to ensure selected CCNTs had different organisational and population characteristics as well as different rates of GP referral (see Table 1).

**Table 1:** Case Study Population and Service Characteristics

		**Case Study**		
**PCT Population**		**A**	**B**	**C**
Population^1^	<15 years old, n (%)	34,300 (18.7)	45,100 (18.1)	38,000 (17.2)
Deprivation^2^		17.9	34.5	45.9
Child Well-being^3^		7.5	20.4	45.8
**Local Healthcare Economy**				
Services				
Hospital(s)		District General	District General	Tertiary Children’s (closed mid-2009), District General
ED		Yes	Yes	Yes (combined)
OAU		No	Yes	
Walk-in Centre (n)		Yes (2)	No	Yes (2)
Emergency Admission Rate at Local Hospital^‡4^		42.3	52.3	57.7
ED attendance rate at Local Hospital^‡5^		376.2	385.1	386.2
GPs per 100,000 children <15^6^		306.4	295.2	416.9
**CCNT**				
Base (Organisation)		Community (PCT)	Hospital (Acute Trust)	Community (PCT)
Number of years established at beginning of study		3	14	2
Disease focus		Acute and Chronic	Acute (and End of Life)	Acute (separate complex needs team)
Referrals^7^	n	923	3,024	3,930
Source (%)	GP	7.3	16.0	2.0
	GP out-of-hours	0.2	-	-
	Walk-in-Centre	5.2	-	0.7
	Parent/Carer	2.1	1.5	1.8
	ED	1.1	26.0	86.7
	OAU	-	15.8	
	Ward	77.0	35.2	-
	Other	7.1	5.5	8.8
Workforce^8^ FTE	n	13.8	14.4	10.0
	per 1,000 children <15	0.40	0.32	0.26
Hours of operation		Mon to Fri 08:00 to 20:00; Sat/Sun/Bank Holidays 08:00 to 18:00	Mon to Sun 08:00 to 20:00	Mon to Sun 08:00 to 22:00
CCNT referral rate per 1,000 children <15^9^		26.9	67.1	103.4

Notes:

^‡ ^Local Hospital is defined as the hospital to which the greatest percentage of children resident in the PCT attend.

Data sources:

^1 ^Office for National Statistics (ONS) mid-year estimates 2008.

^2 ^Percentage of people living in the most deprived quintile of the Index of Multiple Deprivation 2007 (England average: 19.9%) (Source: APHO and DH Health Profile 2009).

^3 ^Percentage of Lower Super Output Areas in Lowest quintile of the national distribution (Source: Local Index of Child Well-being 2009).

^4 ^Emergency Admission Rate for three commonest medical presentations at EDs (i.e., breathing difficulty, feverish illness, diarrhoea) per 1,000 children aged 0–14 resident in the study area (Source: Hospital Episode Statistics 2006/07).

^5 ^ED attendance rate per 1,000 children aged 0–14 registered with a GP in the study area (Source: North West Strategic Health Authority Tactical Information Service 2007/08).

^6 ^GPs per 100,000 children aged 0–14 (Source: The Information Centre for Health and Social Care 2009).

^7 ^Annual Referrals (Source: CCNT routinely collected data 2009).

^8 ^Full-time Equivalent workforce (Source: CCNT A, December 2009; CCNT B, March 2010; CCNT C, December 2009).

^9^ CCNT referral rate per 1,000 children aged 0–14 (Source: CCNT routinely collected data 2009; ONS mid-year estimates 2009).

### Sample

Thirty-nine health professionals were interviewed across the case studies between June 2009 and February 2010. Individuals were purposively sampled to provide a wide range of views from services in each PCT and included: GPs; CCNs; consultant paediatricians; service managers and commissioners.

### Data collection

Semi-structured face-to-face interviews were conducted with participants. Each interview lasted an hour and was conducted in the interviewee’s workplace. Discussion was structured by the following topic guide that included questions about relationships and referral between GPs and CCNTs:

· Individual’s roles, responsibilities and professional background;

· Views around the current and future role of the CCNT;

· Perceived impact of the development of CCN services on roles, relationships, skills, training needs and the local healthcare economy;

· Impact of past, current and future service reconfiguration;

· Impact of the extension of the CCNT’s role in acute care on work with children and young people with long-term conditions;

· Referrals to other agencies such as social care, child protection and education.

### Data analysis

Interviews were audio recorded and transcribed verbatim. Data were managed using NVivo (Version 8). Thematic analysis was conducted using principles and procedures of the Framework approach [15]. First, transcripts were allocated to four co-authors (RGK, MB, SK, PC) to ensure that each was read by two co-authors. During this familiarisation process each reader identified and noted key themes that emerged from the data from their initial independent reading of transcripts. Second, two ‘data workshops’ involving four co-authors (RGK, MB, SK, PC) were convened during which each transcript was discussed in turn to develop an agreed thematic framework to be subsequently applied to the data corpus. Discussion during these data workshops was guided by core research questions; hence this process identified the five factors found to be facilitative of GP referral to CCNT reported in this paper. Third, all transcripts were entered into NVivo and indexed by two co-authors (RGK, MB). Inter-coder reliability was assessed on a 5% sample of indexed data and revealed no substantial differences in the application of the thematic framework between researchers.

### Ethical approval

Ethical approval for the study was obtained from university and local NHS research ethics committees. Interviewees provided informed written consent. Interviewee anonymity is maintained in this paper through the identification of participants by a case study letter (i.e., A, B or C) and interviewee number (e.g., C9).

## Results

### Service Profiles

CCNTs routinely collected activity data identified that they received a minority of referrals from GPs (range 2% to 16%). Referral patterns varied widely, reflecting differences in service design, organisational location, workforce and operation (Table 1). CCNT A was mainly a follow-up service from the ward which was the source of 77% of its referrals; 7% of referrals were from GPs. CCNT B was integrated at multiple points in the urgent care pathway receiving 26% of its referrals from Emergency Departments (ED), 15.8% from an Observation and Assessment Unit (OAU), although its greatest proportion of referrals was also from the ward (35.2%), it had the highest proportion of GP referral (16%). CCNT C was closely integrated with a local ED/OAU facility established as a result of the closure of a children’s hospital in the area. CCNs rotated through this facility which accounted for 86.7% of referrals to the CCNT. CCNT C had the lowest proportion of GP referrals (2%) (Table 1).

### Facilitators of GP referral to CCNTs

Five individual-, organisational- and system-level factors were found to facilitate GP referral to CCNTs:

1. CCN/CCNT visibility (individual-/organisational-level)

2. Clear clinical governance procedures (organisational-level)

3. Financial and organisational investment (organisational-level)

4. Access and out of hours availability (system-level)

5. Facilitative financial frameworks (system-level)

#### CCN/CCNT visibility

Respondents suggested that the visibility of individual CCNs, and the CCNT, facilitated referral from GPs. The development of trusting relationships could encourage GPs to confidently refer children to CCNT care:

‘Quite often you will find that it’s a personal touch that makes the difference. We know that there’s a history of GPs not liking to fill in referral forms and when you’ve worked in primary care as long as I have you very much know that it’s about visibility, that relationship and the trust’. (A8: Senior Nurse)

Each service had conducted a series of presentations to GP practices to increase visibility of the CCNT and awareness of the CCN role in the care of acutely ill children with varying levels of success. In CCNT B practice presentations were supported by a referral protocol that initially restricted permitted referrals to ‘five categories’ comprising the most common conditions in two pilot areas. One area was identified by the CCNT and the other allocated by children’s commissioners as it was the source of the highest proportion of repeat ED attenders – so-called ‘frequent fliers’ – in the area. The aim was to demonstrate CCNs’ clinical competence in these exemplar conditions and so gain the confidence of GPs:

‘We felt that we could get [GPs] engaged and they could see that the clinical confidence was there, they could see that those children were managed.

[…] By starting slow, although it was frustrating because you do want to get all GPs on board, you’ve got to realise that you need to show, you need to be able to demonstrate that we [CCNTs] can make a difference. And it’s not just making a difference; it’s actually proving that those children, although it’s a different route, it’s just as good an outcome – and sometimes better’. (B9: Senior Nurse)

Subsequently, referrals were accepted across a wider area and range of conditions. Most referrals were for self limiting or low risk conditions:

‘We opened it up then to all [GPs], of which […] we’ve got [over a hundred] and we’ve still only got 40-odd using us, but that is a massive increase. We’ve got nearly 200 referrals now from GPs a month which is brilliant’. (B9: Senior Nurse)

Making the CCNTs visible involved presentations to ‘market’ the CCNT to GPs but ‘word of mouth’ communication about the experiences of GPs and of parents themselves was seen as most effective:

‘The CCNT put quite a lot of effort […] coming round to the practices to try to speak to people. But different practices take that in different ways. […] From the GPs’ perspective sort of organisation self-promoting, people might be a bit more sceptical. Whereas if there’s sort of promotion from the service user end it might be “oh, well, maybe yes, well, hadn’t thought about it like that, we’ll give it a go”. And then when you find that it’s working it sort of then becomes self-promoting’. (B2: Hospital Doctor)

‘The parents will ring up and a classic line is: “oh you’ve looked after my friend’s child and oh, it’s been amazing what’s happened and they, they’ve really sort of been talking about your team so how can we have you?”. ’ (B9: Senior Nurse)

Promoting the CCNT from GPs could lead to questions about whether referrals were appropriate:

‘And, yes, they’re common conditions; yes, they’re your feverish illnesses; yes, they’re viral URTIs; yes, they’re children who are constipated – but they’re still having their needs met now in the community rather than having to access secondary care services. So, ok, we might think that it’s just the ‘bread and butter’ but for those families it makes a huge difference’. (B9: Senior Nurse)

This comment highlights the concerns that the CCNT could be receiving referrals for minor conditions that did not require a nursing service. The CCNTs were aware that their funding depended on whether they were seen to be cost effective, which depended on judgements about whether the children referred to them would otherwise have attended hospital. Approaches from parents who had heard about the CCNT and asked for care further highlighted the role of the GP in establishing whether CCNT input was an appropriate use of resources:

‘To be honest a lot of our [self referral] phone calls can be round things like constipation or maybe atopic eczema […] if you weren’t careful you could end up having all children and that’s not what the service is there for. So it’s very important as well that they do go to a professional, that can at least start the journey and that’s not to say that, we don’t want the GPs to do an awful lot, other than refer’. (B9: Senior Nurse)

#### Clear clinical governance procedures

The development and demonstration of clear clinical governance processes within and between organisations was important for GPs to have confidence to refer acutely ill children to the CCNT.

‘The [CCNT] can take direct referrals from the GPs and they’ve got the back-up of either the GP or they can always come to the hot week consultant if what they’ve seen from the GPs isn't quite what they expect they can come straight back to us’. (B1: Hospital Doctor)

Follow-up by the CCNT could therefore also provide reassurance that deteriorations in children’s conditions would be detected:

‘A lot of GPs I think get a bit stuck on where to go next. They may not want to admit the child, but they may not want to leave it 48 hours before it’s next seen, so we’re a good stop gap there, we can go out and reassess and go from there’. (A4: Nurse)

In case study B clinical governance was supported by the organisational location of the CCNT in an acute trust, co-located with the children’s ward and OAU in which children could be assessed without admission for up to 6 hours. This was observed to provide a 'safety net' (B1) as CCNs could rapidly refer children to the OAU from which an onward admission could be secured, where appropriate.

‘GPs are able to refer because I think we’ve proved that our pathways and our governance structures are in place now, we’re bedded in, as in that we’ve got a proper pathway that the GPs refer to us and if that nurse that visits that child feels that they need a paediatric opinion then they come to the OAU and that’s working very well’. (B9: Senior Nurse)

Integration of CCNTs in secondary care could therefore provide the confidence of a ‘safety net’ of consultant cover which enabled GP referral.

#### Financial and organisational investment

Financial and organisational investment in the role of the CCNT in acute care pathways was regarded as a prerequisite for development of GP referral to CCNTs. In each CCNT attempts had been made to increase referrals through the delivery of presentations to GP practices and demonstrating competence through encouraging GPs to refer for a restricted range of common childhood conditions. However the relative success of CCNT B, as demonstrated by a higher GP referral rate, was secured through financial investment in secondments of the CCNT manager to conduct practice presentations and a GP to promote the CCNT among peers. This appointment was considered particularly valuable as an advocate for the CCNT with ‘insider’ status among GP colleagues.

#### Access and out of hours availability

Organisational location and process, particularly in areas where CCNTs were provided by acute trusts, were observed to potentially work against GP referral as highlighted by one GPs’ frustration in working in a system that required referral to the CCNT via a hospital paediatrician:

‘When I [moved] here I knew there were CCNs but they were hospital-based and I couldn’t access them directly so that meant that in order to get a child seen by a community nurse I had to refer through a hospital paediatrician and I just felt that was quite frustrating’. (B3: GP)>

GPs who referred to the CCNT also emphasised the importance of out of hours availability:

‘When you’re trying to do a busy surgery and you’re trying to refer a child, […] I can ring them through and pre-warn them about this fax that will come first thing in the morning so they can already sort of allocate, they’ll take it over the phone. So the fact that you can get ahold of people easily I think is really quite useful’. (B3: GP)

#### Facilitative financial frameworks

Commissioners commented that GP referral has potential to deliver cost savings of ‘somewhere between £60 and £100’ (A9). However, cost effective transfer of work to the community was contingent on facilitative financial frameworks. Commissioners suggested that ‘perverse’ (C12) financial incentives in the acute sector offered ‘no incentive, other than a willingness to ensure care is delivered as close to home as possible, for an acute setting to actively support [CCNTs] in the management of children in the community’ (A8). Activity-based payment systems (e.g., Payment by Results [PbR]) were observed to work against clinically effective care in the community, such as the use of secondary care as a ‘safety net’ (B1) in support of community care:

‘I’m interested in what we could do around the observation and assessment type function of getting a swift paediatric opinion, that would be good use of that resource but without it costing us a full tariff. At the minute we can’t market up the OAU as a way of getting a paediatric opinion because we get charged the full tariff but actually sometimes it would be quite nice to have a small baby admitted, checked over and straight back home again with the [CCNT], and GPs would be very reassured by that’. (B11: Commissioner)

Financial frameworks were found to introduce particular ‘tensions’ for CCNT B because its location in an acute trust meant that ‘if they work well they’re taking business out of the hospital’ (B11). Moreover, local financial agreements could introduce conflict between cost- and clinical-effectiveness:

‘Because of the financial framework we have agreed that we will pay, say a full in-patient tariff in the short-term for any admission to an OAU so it actually is the same amount of money if they’re only admitted to an OAU and then go home. So in the new financial climate it makes no odds to us if they don’t go home from ED we’re stung for the same amount of money regardless. […] If they’re admitted to OAU and then they go home we have paid twice for a service whereas if they’d gone to OAU and then been admitted it would only have cost us one tariff. But if they go to OAU and then go home and see CCNT we’ve paid the full in-patient tariff and then we’ve paid for the care separately. So, in effect, it’s more expensive now than if they were admitted to hospital and that’s one of the financial realities’. (A9: Commissioner)

## Discussion

Attempts to encourage GP referral had met with varying levels of success across the three CCNTs. There was widespread recognition that referral to CCNTs had potential to avoid unnecessary attendances at ED and even hospitalisations, and there was willingness among GPs to refer acutely ill children to CCNT care. Access to a secondary care ‘safety net’ of consultant cover or rapid OAU referral could help give GPs confidence to refer children to the CCNT. Frequent demonstration of clinical competence and decision-making was also important and could lead to ‘word of mouth marketing’ of the CCNT among parents and GPs. In this way trust between GPs and CCNs might be built which may encourage GPs to view CCNTs’ as part of the primary care team which already comprises other community nurses (Practice Nurses, District Nurses), midwives and health visitors, and thereby increase referral rates. However, ensuring the appropriateness of referrals was a continuing concern and GPs were seen to have an important role in identifying children for whom CCNT care would be of additional benefit and could reduce the need for hospital attendance.

The relative success of CCNT B in securing GP referrals suggests that an incremental approach to encourage GP referral is likely to result in increased GP referral rates. This development needed to be underpinned by financial and organisational investment in the role of CCNTs in acute care pathways and recognition that developing GP referral is potentially time-consuming and resource-intensive. However, organisational efforts are contingent upon system-level financial frameworks that reward rather than penalise the transfer of care to community providers.

The low rates of referral to CCNTs C and A highlight the difficulty of encouraging GP referrals. The development of GP referral is likely to be most successful through a combination of the five identified facilitators and thus concerted effort at individual-, organisational-, and system-levels.

Previous research examining primary care integration has tended to focus on the effectiveness of follow-up by primary care physicians after ED attendance or hospitalisation to reduce re-attendance or readmission [16-19], rather than the potential impact of GP referral on avoidable attendance or admission. Studies have found conflicting evidence on the effectiveness of primary care follow-up in the reduction of subsequent use of urgent care services. A randomised controlled trial found that follow-up phone calls from primary care reduced subsequent ED use [18], although another found no association between primary care follow up and subsequent ED use for common childhood conditions [17]. There is evidence of an association between prior ED use and subsequent ED use [17] suggesting that intervention early in an episode of acute illness to avoid initial ED use – such as through GP referral to CCNTs – may prevent subsequent ED attendances or hospital admissions. In common with previous research [20], our findings suggest that there is support for GP referral to CCNTs among professionals, subject to certain safeguards. A study examining GPs’ views of the implementation of a new hospital-at-home service in Rugby, England, found that GPs required clarification around clinical governance to confidently refer acutely ill children to the services [20]. Our own findings identify the importance of the clinical governance ‘safety net’ of onward ED/OAU referral and consultant cover to GP confidence to refer to CCNTs.

GPs expressed a willingness to refer acutely ill children to CCNTs, borne out by both routinely collected referral data and GPs’ comments. However, this was contingent upon referring clinicians’ confidence in CCNs’ clinical competence to safely manage the care of acutely ill children. CCNT intervention earlier in children’s acute care pathways to enable ‘secondary care bypass’ is therefore predicated on frequent demonstration to GPs of the management of clinical risk, particularly for those children whose condition quickly deteriorates requiring rapid onward referral to an OAU or emergency hospitalisation. There are therefore training implications for CCNTs and GPs receiving and making referrals early in children’s contact with the urgent care system, particularly around clinical governance protocols across healthcare providers, not least because of the consequences of missing serious problems. Our findings about the importance of individual-level factors, including CCNT visibility, to establishing GP confidence suggests there is merit in developing joint training programmes between CCNTs and GPs in future. Training should also involve hospital-based paediatricians to further strengthen relationships between children’s services in primary and secondary care.

Development of GP referral was found to be facilitated by the fostering of trustful relationships between GPs and CCNs, requiring financial and organisational investment. However, these relationships take time to mature [21]. Thus, where such relationships are absent or nascent alternative clinical strategies such as ‘secondary care gatekeeping’ by embedding or rotating CCNs in ED/OAU [22] may be required in combination with incremental efforts to encourage ‘secondary care bypass’ through GP referral to deliver reductions in preventable admissions.

Further research is therefore required to examine if and how the extent and nature of GP referrals change with specific attempts by individual CCNTs to develop referral pathways. Investigation into the impact of system-level NHS reforms, particularly proposed changes to underlying financial frameworks involving ‘unbundling tariffs’ and the development of currencies for community and other services [23], is also warranted.

### Strengths and limitations

Despite existing research on the development, design and distribution of CCNTs across England derived largely from small-scale single-service evaluations or national surveys [14,24-27], there has been only limited examination of CCNT integration with secondary [22] and primary care [20]. To our knowledge, this is the first study to identify facilitators of GP referral to CCNTs. The comparative case study design provided a method where ‘complex phenomena can be understood’ [28] within and across CCNTs. This approach allows the views of different professionals located in several organisations to be elicited to arrive at an overall appreciation of how services interact within the wider healthcare economy in a way that single service evaluations or national surveys of only CCNTs could not capture. Hence, our findings identify key facilitators that may transfer to other settings in the UK and internationally to inform the development of referral between primary care physicians and community children's nursing services. However, it should be acknowledged that these findings are derived from a relatively small sample of healthcare professionals (n = 39) and that the limited evidence around the clinical- and cost-effectiveness of CCNTs may influence GPs’ judgements on whether and how to use CCNTs.

## Conclusions

Our comparative study has identified that in order to facilitate referral between GPs and CCNTs GPs required confidence in CCNs’ competence to safely manage acutely ill children at home. GP confidence in CCNs ability to safely manage children at home was secured through frequent demonstration of clinical skills and a ‘safety net’ of rapid access to observation and assessment facilities or paediatric consultant cover if a child’s condition deteriorated. Incremental approaches to developing GP referral to CCNTs underpinned by clear clinical governance protocols are likely to be most effective in building GP confidence and avoiding inappropriate admission.

## Competing interests

The authors declare that they have no competing interests.

## Authors’ contributions

RGK conducted data collection and analysis, drafted and revised the manuscript. MB conducted data collection and analysis. SK secured funding, conducted data analysis and revised the manuscript. PP secured funding and revised the manuscript. PC secured funding, conducted data analysis, drafted and revised the manuscript. All authors approved the final version.

## Pre-publication history

The pre-publication history for this paper can be accessed here:

http://www.biomedcentral.com/1471-2296/14/4/prepub
